# Direct and Indirect Parental Support for Meeting Physical Activity Recommendations Among Chilean Children and Adolescents

**DOI:** 10.3390/bs16091476

**Published:** 2026-08-25

**Authors:** Iván Vallejos Cortés, Katherine Espinoza Valdivia, Natalia Quijada-Mateluna, Johan Rivas-Valenzuela, Carlos Jorquera-Aguilera, Fernando Rodríguez-Rodríguez

**Affiliations:** 1 Escuela de Nutrición y Dietética, Facultad de Medicina, Universidad Mayor, Santiago 8580745, Chilekatherine.espinozav@mayor.cl (K.E.V.); carlos.jorquera@mayor.cl (C.J.-A.); 2 IRyS Research Group, School of Physical Education, Pontificia Universidad Católica de Valparaíso, Valparaíso 2340025, Chile; 3 Group EFyM, School of Physical Education, Pontificia Universidad Católica de Valparaíso, Valparaíso 2340025, Chile; johan.rivas@pucv.cl; 4 Center for Interdisciplinary Research in Biomedicine, Biotechnology and Well-Being (CID3B), Pontificia Universidad Católica de Valparaíso, Valparaíso 2340025, Chile

**Keywords:** school, students, father, mother, active behaviour

## Abstract

Introduction: Physical activity (PA) is vital during development, yet compliance with the 60-min daily moderate-to-vigorous (MVPA) recommendation remains low in Chile. This study examined direct and indirect support reported by mothers and fathers and their separate associations with children’s and adolescents’ compliance with MVPA recommendations. Methods: A cross-sectional study was conducted with 246 schoolchildren (ages 9–19) from three cities. PA was assessed via the YAP questionnaire, while parental support was measured through a 5-item scale. Logistic regression models identified associations with meeting PA guidelines. Results: Only 32.1% of students met PA recommendations, with significant gender disparities (boys 47.7% vs. girls 15.3%). Two paternal instrumental support behaviours were significantly associated with meeting MVPA recommendations: providing transportation (OR = 1.52) and financial support during weekdays (OR = 3.36). None of the maternal support behaviours examined showed statistically significant associations. Conclusions: Paternal logistical and financial contributions are decisive for children’s PA, whereas mothers reported several support behaviours more frequently than fathers. Paternal transportation and weekday financial support were positively associated with meeting MVPA recommendations. Longitudinal studies are needed to determine whether these forms of support contribute prospectively to children’s and adolescents’ PA.

## 1. Introduction

Physical activity (PA) is linked to a wide range of physical and psychological benefits for children and adolescents (Marker et al., 2018). PA is classified as any bodily movement produced by the contraction of skeletal muscles that increases energy expenditure above the resting metabolic rate and is characterised by its modality, frequency, intensity, duration, and practice context (Caspersen et al., 1985). Accordingly, international PA recommendations suggest that children and adolescents should engage in at least 60 min of daily moderate-to-vigorous physical activity (MVPA; Bull et al., 2020).

At the global level, current reality deviates from World Health Organization (WHO) recommendations, as 81% of young people aged 11 to 17 do not meet the established guidelines (Guthold et al., 2020). In Europe, data from 2016 indicated that less than 50% of this group reaches the recommended levels (Van Hecke et al., 2016). Specifically, among 11-year-olds, countries such as Italy (13%), Denmark (15%), and Greece (16%) show the lowest prevalence of compliance, while Finland (41%) and Ireland (38%) report the highest (Inchley et al., 2016). In the United States, only 20% of adolescents meet aerobic and muscle-strengthening guidelines (U.S. Department of Health and Human Services, 2018). In Chile, more recent estimates suggest the outlook is equally critical; PA levels are alarmingly low, with an average of only 37% of children and adolescents reporting being physically active for 60 min at least four days a week (Aguilar-Farias et al., 2024).

Designing effective programs and interventions requires an understanding of the factors associated with PA (Saunders et al., 2024), with the family being considered the most crucial environment (Golan, 2006). In this sense, Social Cognitive Theory (Bandura & Walters, 1977) postulates that individuals acquire behaviours by observing and imitating others. Bandura notes that sports practice serves as a significant source of role models, suggesting that parental PA influences that of their children through observational learning (Carter et al., 2022). Within Social Cognitive Theory, behaviour is understood as the product of reciprocal interactions among personal, behavioural, and environmental factors (Taymoori et al., 2010). Parental support may contribute to children’s PA by modifying the immediate social and environmental conditions in which participation occurs. Instrumental behaviours, such as providing transport or paying activity-related costs, may increase access and reduce practical barriers (Timperio et al., 2013). Motivational and informational behaviours, including encouragement and discussion of the benefits of PA, may reinforce positive outcome expectations and perceived social approval. Co-participation may also provide opportunities for observational learning and behavioural reinforcement (Beets et al., 2010). However, because the present study did not directly assess self-efficacy, modelling, outcome expectations, or observational learning, Social Cognitive Theory is used as an interpretative framework rather than as a directly tested theoretical model. Social Role Theory may additionally help contextualise differences in the frequency and type of support reported by mothers and fathers, as caregiving, financial, and logistical responsibilities can be unequally distributed within families. Nevertheless, whether similar support behaviours have different associations according to parent sex remains an empirical question. In this framework, behavioural modelling and parental support toward PA practice constitute a fundamental influence during childhood (Gerards et al., 2021).

Various studies have examined how parental support influences PA. The ENERGY study (Timperio et al., 2013) identified financial, logistical, and emotional support, as well as co-participation, highlighting the relevance of the time parents dedicate to PA (van Stralen et al., 2011). Similarly, research among Chinese schoolchildren identified dimensions that benefit MVPA compliance, including accompaniment and knowledge sharing (Liu et al., 2017). Parental support may be associated with PA through several complementary pathways (Hosokawa et al., 2023). Transportation and accompaniment can reduce geographical, safety, and scheduling barriers to accessing organised activities (Savolainen et al., 2024). Financial support can facilitate enrolment fees, equipment acquisition, and transport costs. Co-participation can provide opportunities for reinforcement and shared activity, whereas encouragement and communication about the benefits of PA may increase perceived approval, motivation, and positive outcome expectations (Smith & Côté, 2024). These mechanisms may vary according to the child’s age, sex, socioeconomic circumstances, and availability of local activity opportunities.

However, there is a notable lack of evidence in Latin American contexts, where socio-environmental and gender barriers may modulate parental support differently than in the Northern Hemisphere. In Chile, factors such as financial support and transport logistics can be decisive (Xu et al., 2015). These differences may be partly explained by social role theory (Eagly & Wood, 2016), which posits that the historical division of labour between men and women generates distinct behavioural expectations that persist within family contexts (Eagly & Koenig, 2021). Consistent with this framework, international evidence shows that parents often take differentiated roles in supporting children’s PA according to traditional gender expectations, even as these roles have been gradually shifting (Roles of mothers and fathers). In the Chilean context specifically, mothers and fathers have been shown to adopt differentiated roles according to cultural gender mandates (Salas et al., 2018). Operationally, we will understand direct parental support as parental behaviours involving active participation or immediate involvement in the child’s PA, such as co-participation, transportation, supervision, or accompanying the child to activity settings and indirect parental support as behaviours that facilitate or encourage participation without the parent necessarily participating in the activity itself, including verbal encouragement, communicating the importance or benefits of PA, and providing financial or material resources. Furthermore, Chilean studies have shown that family support is positively associated with active lifestyles and participation in PA, reinforcing the need to examine maternal and paternal contributions separately within this specific sociocultural context (Aguilar-Farias et al., 2024; Pinheiro et al., 2026; Rodríguez-Rodríguez et al., 2026; Salas et al., 2018).

To our knowledge, no previous Chilean study has simultaneously examined maternal and paternal direct and indirect support separately in relation to compliance with MVPA recommendations. Based on previous evidence suggesting that parental support is positively associated with children’s PA, we hypothesised that both maternal and paternal support would be positively associated with meeting the recommended levels of moderate-to-vigorous physical activity (MVPA). Furthermore, we expected that the strength of these associations would differ according to the parent providing the support. Therefore, this study aimed to examine maternal and paternal direct and indirect support separately and their associations with meeting MVPA recommendations among Chilean children and adolescents.

## 2. Methods

### 2.1. Study Design

The ACTIve BEhaviour in School Education project (ACTIBESE), funded by the National Agency for Research and Development (ANID) of the Chilean government under code number 1230801, aims to explore effective strategies for enhancing PA levels in schools. This three-year project comprises a cross-sectional study in the first and second years, followed by an intervention study in the third year (Rodríguez-Rodríguez et al., 2025). The intervention study was registered on 8 April 2024, at https://clinicaltrials.gov/study/NCT06362655 (accessed on 25 August 2025) with the identifier NCT06362655. However, the findings reported in this paper are based solely on the cross-sectional phase of the project.

### 2.2. Recruitment

Invitations were sent to the principals of participating schools, inviting them to join the project. Upon their agreement and selection, a meeting was scheduled with representatives from the administrative management authorities and schoolteachers. During this meeting, the aims and stages of the project were explained in detail.

Following this, an informational session was conducted for parents and students, categorised by age groups: children from grades 4 to 6 of primary school (9–11 years old) and adolescents from grade 7 of primary school to grade 3 of secondary school (12–19 years old).

### 2.3. Participants and Sample Selection

The participants were selected from two cities in the Valparaíso Region (Viña del Mar and Los Andes) and one city in the Araucanía Region (Temuco). These cities were chosen for the convenience of researchers due to their proximity to the institutions conducting the study in Chile. Participants were recruited through non-probabilistic convenience sampling. The sample included public, subsidised, and private administration schools, as schoolchildren in Chile are typically categorised into these three groups. A total of 152 schools were identified across Viña del Mar (84 schools with 25,176 students), Los Andes (19 schools with 7401 students), and Temuco (49 schools with 21,647 students) (source: https://reportescomunales.bcn.cl/, accessed on 30 March 2022). For this cross-sectional part, a target sample of approximately 382 students was initially estimated using a 95% confidence level, 5% precision, and 50% expected prevalence. To reach this, 500 eligible students were invited; 275 provided parental consent. After exclusion, 246 students with valid PA and parental support data were included in the present analysis. Therefore, the analytical sample should not be considered statistically representative of the total school population.

The sample of schoolchildren was established using cluster sampling: 85 from Viña del Mar, 145 from Los Andes, and 16 from Temuco. Each schoolchild was required to have a father, a mother, or both available to complete the questionnaire. In total, 315 parents responded to the questionnaire. For each participating child or adolescent, volunteer parents (either the mother, the father or both) were invited to complete the parent questionnaire. Therefore, mothers and fathers represent independent subsamples rather than paired parental responses.

School inclusion criteria required that participating schools offer at least two Physical Education and Health (PEaH) classes per week, specifically in the selected grades (from 4th grade of primary to 3rd grade of secondary). Importantly, schoolchildren, parents, or guardians must not concurrently participate in other programs that promote PA.

### 2.4. Instruments for Evaluating Interpersonal Factors

#### 2.4.1. Sociodemographic Variables

A multicomponent questionnaire was used to collect information on sociodemographic and PA variables. Sociodemographic parameters included gender, age, school grade, and socioeconomic status, which was assessed using the Family Affluence Scale (FAS III). This scale considers aspects such as ownership of vehicles, household appliances, electronic devices, bathrooms, individual bedrooms, and vacation destinations (Torsheim et al., 2016). Based on the score obtained, socioeconomic status is classified as low (1–4 points), medium (5–8 points), or high (9–12 points).

#### 2.4.2. Physical Activity in Children, Adolescents, and Parents

PA in children and adolescents was assessed using two instruments. The Youth Activity Profile (YAP) questionnaire measured MVPA and sedentary behaviour. The YAP-SL consists of 15 items divided into three sections: Activity at School, Out-of-School Activity, and Sedentary Habits. Scores range from 1 to 5, reflecting increasing levels of PA. The YAP questionnaire showed moderate-to-high reliability in Chilean children and adolescents, and a good quality was applied in this population (Brand et al., 2024). In addition, the YAP scores were converted to minutes/day of MVPA using Fairclough’s equations (Fairclough et al., 2019). A daily MVPA threshold of >60 min/day is considered “physically active,” while those below this threshold are classified as “physically inactive.”

#### 2.4.3. Parental Support

Parental support was assessed using a 5-item scale measuring perceived paternal and maternal support. Participants rated the frequency with which parents engaged in activities that encourage PA and provided support for such activities. This included aspects such as providing transportation, observing and praising PA, and participating in physical activities with their children (Prochaska et al., 2002). The Parental Support for PA Scale demonstrated acceptable reliability, with Cronbach’s alpha coefficients exceeding 0.70 in the overall sample as well as in analyses stratified by age and sex. In addition, the instrument exhibited strong internal consistency, and four of its five items showed moderate to high reliability (κ = 0.50–0.69) across all analysed subgroups within the Chilean population (Brand et al., 2025).

### 2.5. Data Analysis

Data normality was assessed using Q-Q plots (quantile-quantile plots), and the interaction between sex and age was examined. Descriptive statistics for study variables were calculated, including means and standard deviations for continuous variables and frequencies and percentages for categorical variables. To establish the association of dichotomous variables, binary logistic regression models were performed to obtain the odds ratio (OR) with their respective confidence intervals (95% CI). Analyses were conducted using chi-square tests for categorical variables. Separate univariate binary logistic regression models were performed for mothers and fathers because parental responses represented independent subsamples rather than paired observations. Each parental support variable was entered individually to estimate its association with meeting the MVPA recommendations. No additional covariates were included because the purpose of the analysis was exploratory and focused on the independent association of each support behaviour; no multivariable adjustment was performed. In the present sample, the Parental Support Scale showed acceptable internal consistency (Cronbach’s α = 0.90 for mothers and α = 0.89 for fathers). Pearson’s chi-square tests were used to compare the distribution of maternal and paternal support responses. Composite scores correspond to the average frequency across the items classified as direct and indirect parental support. A sensitivity analysis of the logistic regression models was conducted (S1) JASP statistical package V 0.18.3 (JASP Team, 2024), a free software interface Version 0.18.3 (Nieuwe Achtergracht 129B, Amsterdam, The Netherlands), was used for analysis. Statistical significance was set at *p* < 0.05.

### 2.6. Ethical Considerations

Following approval from the participating institutions, written informed consent was obtained from the parents or legal guardians of all participating children and adolescents. In addition, all participating students provided written assent before data collection after receiving age-appropriate information regarding the study objectives and procedures. The study was conducted in accordance with the Declaration of Helsinki (World Medical Association, 2013) and was approved by the Ethics Committee of the Pontificia Universidad Católica de Valparaíso (BIOEPUCV-H 638-2023).

## 3. Results

Table 1 presents the sociodemographic characteristics of students and their parents. The sample consists mainly of adolescents (62.6%), with a predominantly middle socioeconomic level (52.0%) and a higher proportion attending public schools (44.5%). Additionally, a large portion of the students do not meet the recommendations for MVPA (67.9%). Regarding the parents, there is a diverse educational level, with a considerable proportion holding a university degree (39.2%) or a professional degree (24.1%).

Table 2 describes the distribution of different parental support behaviours for PA reported by mothers and fathers, expressed as absolute frequencies (N) and percentages (%), together with the corresponding *p*-values for comparisons between parental roles.

Regarding observing their offspring practising PA or sport, all parents reported some level of involvement; however, mothers more frequently reported everyday observation (9.0%) compared with fathers (6.5%), while fathers more often indicated never observing (43.0% vs. 37.9%). These differences were statistically significant (*p* < 0.001).

In relation to providing transport for PA or sport, fathers reported higher engagement in this behaviour, particularly in the sometimes category (74.9% vs. 59.9%), whereas mothers more frequently reported never providing transport (26.9% vs. 18.5%). The differences between mothers and fathers were statistically significant (*p* = 0.001).

For co-participation in PA or sport with their offspring, no statistically significant differences were observed between mothers and fathers (*p* = 0.174). Nevertheless, descriptive data indicate that fathers more often reported sometimes practising PA with their offspring (51.0%), whereas mothers more frequently reported never doing so (61.1%).

Concerning taking and picking up offspring from PA venues during weekends, mothers more frequently reported always performing this role (30.5% vs. 20.4%), while fathers more often selected the sometimes category (52.7% vs. 34.3%). These differences were statistically significant (*p* < 0.001).

With respect to practising PA with offspring during weekends, fathers reported higher participation in the sometimes category (62.0% vs. 51.4%), whereas mothers more often reported never (40.6% vs. 32.4%). The difference between parental roles was statistically significant (*p* = 0.010).

Finally, for practising PA with offspring during working days, no significant differences were found between mothers and fathers (*p* = 0.295). In both groups, the majority reported never engaging in PA with their offspring on weekdays.

Overall, the table highlights distinct parental role patterns, with mothers showing greater involvement in supervisory and logistical support, particularly during weekends, while fathers tend to report higher participation in transport-related support and occasional co-participation in PA.

Table 3 presents the distribution of indirect parental support for PA as reported by mothers and fathers. With respect to encouragement to engage in PA or sport, both mothers and fathers reported high levels of involvement, with similar proportions indicating “Everyday” encouragement (38.7% and 38.0%, respectively). Despite these comparable percentages, the overall distribution differed significantly between parental roles (*p* < 0.001), mainly due to slight variations in the “Sometimes” and “Never” categories.

Regarding communicating the importance of PA or sport, mothers reported higher daily involvement (39.8%) compared with fathers (26.9%), whereas fathers more frequently reported “Sometimes” providing this information (65.7% vs. 50.7%). These differences were statistically significant (*p* < 0.001), indicating a more consistent verbal emphasis on PA among mothers.

In terms of explaining the benefits of PA or sport, mothers again reported higher “Everyday” engagement (44.1%) than fathers (30.6%); although this difference did not reach statistical significance (*p* = 0.063), suggesting broadly similar patterns between parental roles.

Concerning financial support for PA during weekends, mothers more often reported “Always” spending money to support their offspring’s PA (25.4% vs. 19.4%), while fathers more frequently selected the “Sometimes” category (41.7% vs. 31.4%). These differences were statistically significant (*p* < 0.001). A similar pattern was observed for financial support during working days, with significant differences between mothers and fathers (*p* < 0.001), although in both groups nearly half reported “Never” providing financial support on weekdays.

Regarding support for PA that does not involve spending money, mothers more frequently reported: “Always” providing this type of support (40.8% vs. 36.1%), whereas fathers more often reported “Sometimes” (*p* < 0.001), highlighting complementary forms of non-economic involvement.

In relation to accompanying offspring to sport or PA spaces during weekends, mothers reported higher “Always” involvement (26.3%), while fathers more often reported “Sometimes” (48.1%). These differences were statistically significant (*p* < 0.001).

Finally, for encouraging offspring to be physically active during weekends and working days, mothers consistently reported higher “Always” encouragement.

Mothers reported higher frequencies of observing their children during PA and communicating the importance of PA, whereas fathers more frequently reported providing transportation. Statistically significant differences were observed for these variables.

Table 4 shows the associations between direct and indirect support of mothers and fathers and reaching the PA recommendations. Direct support of fathers was only found when they transported their children to do PA or some sport activity. Also, an association of indirect support was shown only once, when fathers spent some money on working days to support their offspring’s practice of PA. No association was found for direct or indirect maternal support.

**Figure 1:**
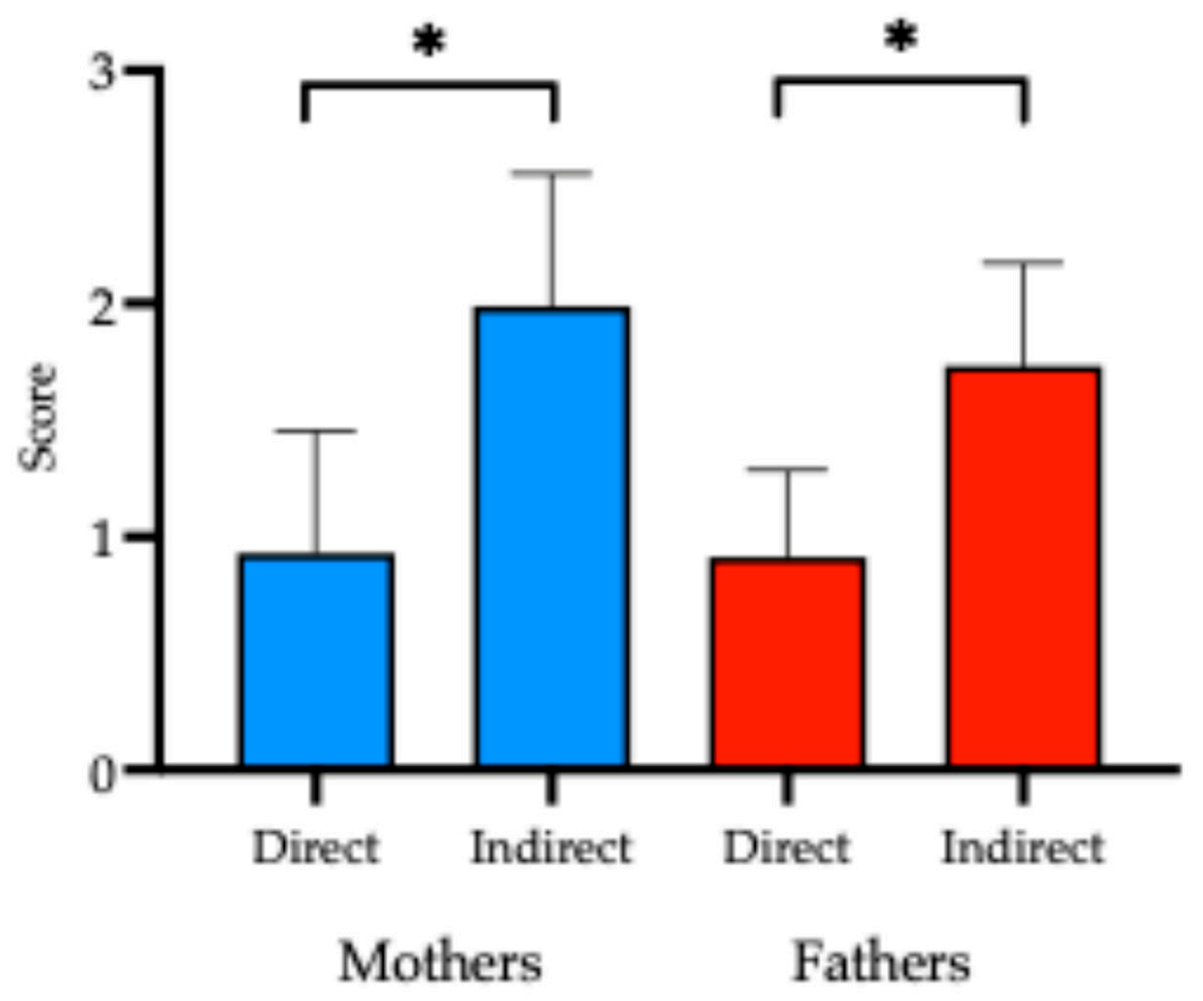
Mean scores of direct and indirect parental supports for PA reported by mothers and fathers. * Statistical difference for *p* < 0.05.

Figure 1 illustrates the mean score values for direct and indirect parental support for PA, as reported separately by mothers and fathers. As detailed in the accompanying statistical analysis, mothers reported significantly lower perceptions of direct parental support (Mean = 0.93) compared with indirect support (Mean = 1.99), with this difference reaching statistical significance (*p* = 0.002). A similar trend was observed among fathers: perceived direct support (Mean = 0.91) was significantly lower than indirect support (Mean = 1.73), also with a statistically significant difference (*p* = 0.002).

Overall, the figure reinforces the quantitative findings by visually demonstrating that, regardless of parental role, indirect forms of support (e.g., logistical or environmental facilitation) are more prevalent than direct forms of support (e.g., co-participation or direct encouragement). This pattern suggests a systematic preference for indirect involvement in supporting children’s PA among both mothers and fathers.

In Table 4, the univariate logistic regression between the levels of support from parents and adherence to the recommended 60 min per day of PA is observed. It is noted that the only statistically significant association was found between direct parental support in transporting their children to engage in PA or sports and adherence to the recommendations. As for indirect support, a statistically significant association was only observed between parents spending money on sports activities during the week and the adherence to PA of their children.

## 4. Discussion

The present findings indicate that two forms of paternal instrumental support—transportation and weekday financial support—were positively associated with meeting PA guidelines among Chilean children and adolescents. In contrast, none of the maternal support behaviours examined showed statistically significant associations with guideline adherence. Given the cross-sectional design, these findings should be interpreted as associations and not as evidence of predictive or causal relationships.

### 4.1. Physical Activity

According to our findings, within a total sample of 246 children and adolescents, only 32.1% achieved PA levels aligned with WHO recommendations. While this compliance is low, it sits above the reported average for adolescents in Latin America, where the figure reaches barely 15% (Guthold et al., 2020). Notable gender disparities were observed; girls showed relatively low adherence (15.3%) compared to boys (47.7%). This gender gap is alarming and consistent with global trends (World Health Organization, 2022). At a regional level, physical inactivity is more prevalent in women (43.7%) than in men (34.4%) (Guthold et al., 2018). An analysis of 146 countries showed that, on average, PA participation was 7 percentage points lower in girls than in boys (Guthold et al., 2020). International research suggests that the most significant barriers for girls’ participation in PA can be mitigated when family and peer values and support are present, along with perceptions of safety in the built environment and opportunities for activity within schools, including quality and inclusive physical education. In the Chilean context, these findings are particularly relevant, as adherence to the WHO recommendation of 60 daily minutes of PA remains low in the national population (Bull et al., 2020). A meta-analysis of 112 studies on parental influence demonstrated that children and adolescents presented higher PA levels when receiving positive parental support (r = 0.202, *p* < 0.001; Su et al., 2022), validating the importance of the parental factor in this population.

### 4.2. Direct and Indirect Parental Support

A central descriptive finding of this study was that indirect support was reported more frequently than direct support by both mothers and fathers. Mean indirect-support scores were higher than direct-support scores among mothers (1.99 vs. 0.93) and fathers (1.73 vs. 0.91). According to the operational definitions used in the present study, this pattern indicates that parental support was more commonly expressed through encouragement, communication, financial provision, and other facilitative behaviours than through behaviours involving immediate parental participation or involvement in the child’s PA. Previous research has similarly shown that parental support is multidimensional and may include encouragement, logistical assistance, transportation, co-participation, financial provision, and modelling behaviours (Beets et al., 2010; Yao & Rhodes, 2015; Su et al., 2022).

Direct support in the present study included behaviours involving immediate parental involvement, such as providing transportation, accompanying the child, observing participation, and engaging in PA together. Among these behaviours, paternal transportation was the only direct-support item statistically associated with meeting MVPA recommendations. This finding is consistent with studies identifying transportation and other forms of logistical assistance as parental support behaviours associated with children’s PA or MVPA (Timperio et al., 2013; An et al., 2021; Hosokawa et al., 2023). Nevertheless, the remaining direct-support behaviours, including co-participation and observation, were not significantly associated with guideline adherence in the present sample. These non-significant findings should not be interpreted as evidence that such behaviours are unimportant, particularly because meta-analytic evidence indicates considerable heterogeneity in associations according to the support behaviour assessed, the characteristics of the participants, and the measurement of PA (Yao & Rhodes, 2015; Su et al., 2022).

Indirect support, as operationalised in this study, included encouragement, communication about the importance and benefits of PA, and financial or material facilitation. Although these behaviours were reported more frequently than direct support, only paternal financial support during weekdays was statistically associated with meeting MVPA recommendations. Therefore, neither direct nor indirect support showed consistent associations across all constituent behaviours. Rather, the findings suggest that specific instrumental behaviours within each category, transportation within direct support and financial provision within indirect support, were associated with guideline adherence. This behavioural specificity is consistent with evidence showing that different forms of parental support may have different associations with children’s PA and should not necessarily be treated as interchangeable indicators of a single support construct (Beets et al., 2010; Yao & Rhodes, 2015; Hong et al., 2020).

These results highlight the value of examining parental support at the behavioural-item level, rather than assuming that all behaviours classified as direct or indirect represent homogeneous exposures. Previous conceptual and empirical work has shown that parental support encompasses distinguishable behaviours that may address different conditions for participation, including access, motivation, companionship, and practical assistance (Beets et al., 2010; Timperio et al., 2013; Hosokawa et al., 2023). Transportation may be particularly relevant when access to PA opportunities depends on distance, scheduling, perceived safety, or the availability of organised activities, whereas financial support may be related to enrolment fees, equipment, or transport expenses (Timperio et al., 2013; Hosokawa et al., 2023). However, these barriers and mechanisms were not directly assessed in the present study. Accordingly, they should be considered plausible interpretations rather than explanations demonstrated by the current results.

Differences between mothers and fathers provide an additional level of interpretation. Mothers reported more frequent verbal and informational support in several domains, whereas fathers more frequently reported some forms of transportation and co-participation. Previous research has also identified differences in the frequency and type of support provided by mothers and fathers, although the direction and magnitude of these differences vary according to the child’s sex, the day of the week, the PA context, and the support behaviour examined (An et al., 2021). Such patterns may be interpreted in relation to differentiated family roles and responsibilities; however, the present study did not directly assess the division of household labour, parental employment, time availability, caregiving responsibilities, or gender-role beliefs. Therefore, interpretations based on Social Role Theory should remain tentative and should not be understood as mechanisms tested by this study.

### 4.3. Support Associated with PA

The regression analyses showed that two paternal instrumental support behaviours were associated with meeting PA recommendations, whereas maternal support did not show statistical significance. It has been found that direct parental involvement through strategies such as providing transportation or supervision fosters greater commitment to regular PA (Su et al., 2022). The observed association suggests that transportation support may represent one of several parental behaviours associated with compliance with MVPA recommendations. However, the magnitude of this association was modest (OR = 1.52), and the confidence interval was relatively close to the null value. Therefore, this finding should be interpreted cautiously and confirmed in future longitudinal studies. This finding aligns with recent research demonstrating that parental instrumental support, such as enrolling children in sports clubs or providing transportation, is significantly associated with higher levels of MVPA during childhood (Hosokawa et al., 2023).

This difference could be explained by traditional gender roles that still prevail in the Latin American context. While maternal support is often perceived as constant care, provided unconditionally regardless of whether the child practices sports, paternal support tends to be more instrumental (Salas et al., 2018). One possible interpretation is that paternal involvement in transportation may reflect greater access to PA opportunities, particularly in contexts where distance, scheduling, or perceived safety constitute practical barriers (Hosokawa et al., 2023; Xu et al., 2015). Nevertheless, these mechanisms were not directly assessed in the present study and should therefore be regarded as hypotheses requiring examination in longitudinal or experimental research. Mothers reported encouragement and communication about the importance of PA more frequently than fathers; however, these maternal support behaviours were not statistically associated with meeting MVPA recommendations in the present analyses. This absence of statistical significance should not be interpreted as evidence that maternal support is ineffective or irrelevant. Maternal support may operate through pathways not captured by the present measure, may be more relevant to other PA outcomes, or may require a larger sample to estimate associations with greater precision. Previous studies have reported heterogeneous associations across different forms of parental support, suggesting that the relationship may depend on the type of support, the child’s age and sex, the PA context, and the measurement method used. Therefore, the non-significant maternal associations observed here should be interpreted cautiously rather than as evidence of no relationship (Hosokawa et al., 2023).

From the perspective of Social Cognitive Theory, it is suggested that simple verbal encouragement is insufficient if not accompanied by environmental modifications (Bandura & Walters, 1977). While encouragement from both parents is positive, paternal financial support during weekdays showed the largest observed association with meeting MVPA recommendations. Providing economic resources may reduce financial barriers to participation in organised PA. It could also be related to psychological constructs such as perceived behavioural control or self-efficacy, although these constructs and potential mediating pathways were not assessed in the present study. Furthermore, perceived self-efficacy acts as a fundamental mediating variable in the relationship between parental support and the practice of PA (Guillén García, 2007). A parent providing economic resources directly relates to the adolescent’s self-efficacy by eliminating the cost barrier, thereby enabling active behaviour (Palma-Quezada et al., 2023). Through instrumental support, parents facilitate the development of beliefs among adolescents regarding their ability to be physically active, which is essential considering that, as Bandura and Walters (1977) point out, those who do not perceive their actions as effective do not initiate them.

Future research should employ longitudinal designs and objective measurements to clarify the causal pathways of parental support. In Latin America, these efforts must account for how family cohesion and shared sedentary habits (such as collective television viewing) are associated with activity levels (Betancourt, 2015; John et al., 2022). This approach is essential for designing culturally pertinent interventions that address the inherent complexity of the Latin American family (Pérez-Flores et al., 2023).

### 4.4. Strengths and Limitations

This study has several strengths. It is among the first conducted in Chile to examine maternal and paternal support separately while distinguishing between direct and indirect support in relation to compliance with moderate-to-vigorous physical activity (MVPA) recommendations among children and adolescents. Additionally, validated instruments were used, and the study was guided by Social Cognitive Theory as a conceptual framework for interpreting the findings.

Several limitations should also be acknowledged. First, the cross-sectional design precludes causal inference and does not establish the temporal direction of the observed associations. Second, physical activity and parental support were assessed using self-reported questionnaires, which may be subject to recall and social desirability bias, while objective measures such as accelerometry were not available. Third, although the analyses explored associations between parental support behaviours and MVPA compliance, residual confounding from unmeasured factors cannot be excluded, and the observed associations should therefore be interpreted cautiously. Fourth, the sample was drawn from three Chilean urban centres, limiting the generalisability of the findings to other geographical and socioeconomic contexts. Finally, although Social Cognitive Theory informed the interpretation of the results, core constructs such as self-efficacy and observational learning were not directly measured. Therefore, future longitudinal studies using objective measures and multivariable analytical approaches are needed to confirm these findings.

## 5. Conclusions

Contrary to expectations, no significant associations were found for maternal support, despite mothers being the ones who most frequently provide verbal encouragement. Two paternal support behaviours, transportation and weekday financial support, were positively associated with meeting MVPA recommendations among Chilean children and adolescents. Higher indirect support than direct support and compromise for PA were found in parents. Given the cross-sectional design, these findings should be interpreted as associations rather than causal relationships. Future longitudinal studies using objective measures of physical activity and multivariable analytical approaches are needed to confirm these findings and clarify the mechanisms underlying parental support for physical activity. Future studies should further investigate the cultural and psychological mechanisms that promote PA, reduce the gender gap in PA levels, and clarify the conditions under which different maternal and paternal support behaviours may be associated with children’s and adolescents’ PA, considering the complex interaction between family, sociocultural, and environmental factors.

## Figures and Tables

**Table 1:** Sociodemographic characteristics of students and parents and physical activity of students.

Students’ Variables	Total		Girls		Boys	
	N	(%)	N	(%)	N	(%)
Sociodemographic	246	(100.0)	118	(48.0)	128	(52.0)
Age (ẋ ± SD)	12.6	±1.8	12.5	±1.9	12.7	±1.8
Age category	246	(100.0)	118	(48.0)	128	(52.0)
Children	92	(37.4)	51	(20.7)	41	(16.7)
Adolescents	154	(62.6)	67	(27.2)	87	(35.4)
Socioeconomic level	152	(100.0)	73	(48.0)	79	(52.0)
Low	58	(38.2)	24	(15.8)	34	(22.4)
Median	79	(52.0)	39	(25.7)	40	(26.3)
High	15	(9.8)	10	(6.5)	5	(3.3)
Kind of School	155	(100.0)	75	(48.4)	80	(51.6)
Public	69	(44.5)	30	(19.4)	39	(25.1)
Subsidized	29	(18.7)	12	(7.7)	17	(11.0)
Private	57	(36.8)	33	(21.3)	24	(15.5)
MVPA (min/day)	246	(100.0)	118	(100.0)	128	(100.0)
No Reach	167	(67.9)	100	(84.7)	67	(52.3)
Reach	79	(32.1)	18	(15.3)	61	(47.7)
**Parents Variables**	**Total**		**Mothers**		**Fathers**	
	**N**	**(%)**	**N**	**(%)**	**N**	**(%)**
Sociodemographic	315	(100.0)	209	(66.3)	106	(33.7)
Age (ẋ ± SD)	42.8	±7.6	41.9	±7.9	44.5	±6.8
Educational level	311	(100.0)	208	(100.0)	103	(100.0)
No studies	4	(1.3)	3	(1.4)	1	(1.0)
Primary school	24	(7.7)	18	(8.7)	6	(5.8)
Secondary school	86	(27.7)	62	(29.8)	24	(23.3)
Professional	75	(24.1)	49	(23.6)	26	(25.2)
University degree	122	(39.2)	76	(36.5)	46	(44.7)

ẋ: mean; ±SD: standard deviation; MVPA: moderate-vigorous physical activity.

**Table 2:** Direct support by sex of the parents for the practice of physical activity or sports in children and adolescents.

Parental Support Topics	Mother		Father		*p* Value
	N	(%)	N	(%)	
Observe the child participating in PA or sport	211	(100.0)	107	(100.0)	
Everyday	19	(9.0)	7	(6.5)	<0.001 *
Sometimes	112	(53.1)	54	(50.5)	
Never	80	(37.9)	46	(43.0)	
Provide transportation to PA or sport	212	(100.0)	108	(100.0)	
Everyday	28	(13.2)	18	(16.7)	0.001 *
Sometimes	127	(59.9)	70	(74.9)	
Never	57	(26.9)	20	(18.5)	
Participate in PA or sport together with the child	211	(100.0)	108	(100.0)	
Everyday	12	(5.7)	5	(4.6)	0.174
Sometimes	70	(33.2)	55	(51.0)	
Never	129	(61.1)	48	(44.4)	
During the weekend I take and pick my offspring from the place where he/she does PA	213	(100.0)	108	(100.0)	
Always	65	(30.5)	22	(20.4)	<0.001 *
Sometimes	73	(34.3)	57	(52.7)	
Never	75	(35.2)	29	(26.9)	
Practice PA with my offspring during the weekend	212	(100.0)	108	(100.0)	
Always	17	(8.0)	6	(5.6)	0.010 *
Sometimes	109	(51.4)	67	(62.0)	
Never	86	(40.6)	35	(32.4)	
Practice PA with my offspring during the working days	212	(100.0)	108	(100.0)	
Always	7	(3.3)	1	(1.0)	0.295
Sometimes	82	(38.7)	46	(42.9)	
Never	123	(58.0)	60	(56.1)	

PA: Physical Activity. * Statistical difference *p* < 0.05.

**Table 3:** Indirect parental support by sex of the parents for the practice of physical activity or sports in children and adolescents.

Parental Support Topics	Mother		Father		*p* Value
	N	(%)	N	(%)	
Encourages you to do PA or sports	212	(100.0)	108	(100.0)	
Everyday	82	(38.7)	41	(38.0)	<0.001 *
Sometimes	117	(55.2)	59	(54.6)	
Never	13	(6.1)	8	(7.4)	
Talk with the child about the importance of PA	211	(100.0)	108	(100.0)	
Everyday	84	(39.8)	29	(26.9)	<0.001 *
Sometimes	107	(50.7)	71	(65.7)	
Never	20	(9.5)	8	(7.4)	
Talk with the child about the benefits of PA	211	(100.0)	108	(100.0)	
Everyday	93	(44.1)	33	(30.6)	0.063
Sometimes	102	(48.3)	66	(61.1)	
Never	16	(7.6)	9	(8.3)	
Provide financial support for PA during weekends	213	(100.0)	108	(100.0)	
Always	54	(25.4)	21	(19.4)	<0.001 *
Sometimes	67	(31.4)	45	(41.7)	
Never	92	(43.2)	42	(38.9)	
Support my child to participate in PA without financial expenditure	213	(100.0)	108	(100.0)	
Always	87	(40.8)	39	(36.1)	<0.001 *
Sometimes	96	(45.1)	55	(50.9)	
Never	30	(14.1)	14	(13.0)	
I accompany my offspring to the sports spaces on the weekend	213	(100.0)	108	(100.0)	
Always	56	(26.3)	26	(24.1)	<0.001 *
Sometimes	81	(38.0)	52	(48.1)	
Never	76	(35.7)	30	(27.8)	
Provide financial support for PA during working days	213	(100.0)	108	(100.0)	
Always	43	(20.2)	22	(20.4)	<0.001 *
Sometimes	70	(32.9)	40	(37.0)	
Never	100	(46.9)	46	(42.6)	
Encourage the child to be physically active during weekends	212	(100.0)	108	(100.0)	
Always	87	(41.0)	39	(36.1)	<0.001 *
Sometimes	105	(49.6)	59	(54.6)	
Never	20	(9.4)	10	(9.3)	
Encourage the child to be physically active during weekends	210	(100.0)	108	(100.0)	
Always	84	(40.0)	35	(32.7)	0.001 *
Sometimes	102	(48.6)	60	(56.1)	
Never	24	(11.4)	12	(11.2)	

PA: Physical Activity. * Statistical difference.

**Table 4:** Associations between direct and indirect parental support by sex of the parents and the reach of PA recommendations for their children.

	Mothers				Fathers			
			95% CI				95% CI	
Direct Support	OR	*p* Value	Lower	Upper	OR	*p* Value	Lower	Upper
Observe the child participating in PA or sport	1.08	0.780	0.780	1.500	0.69	0.181	0.395	1.191
Provide transportation to PA or sport	1.28	0.923	0.923	1.773	1.52	* 0.046	1.008	2.288
Participate in PA or sport together with the child	1.45	0.961	0.961	2.188	0.93	0.809	0.504	1.707
During the weekend pick from the place where does PA	1.01	0.728	0.728	1.404	1.37	0.209	0.838	2.237
Practice PA with my offspring during the weekend	0.70	0.425	0.425	1.154	0.59	0.229	0.249	1.394
Practice PA with my offspring during the working days	0.81	0.474	0.474	1.396	1.14	0.784	0.452	2.865
**Indirect Support**								
Encourages you to do PA or sports	1.10	0.553	0.807	1.494	1.42	0.158	0.874	2.293
Talk with the child about the importance of PA	1.06	0.826	0.653	1.705	0.91	0.826	0.383	2.150
Talk with the child about the benefits of PA	0.92	0.715	0.566	1.477	0.64	0.323	0.266	1.548
Provide financial support for PA during weekends	1.03	0.875	0.695	1.533	0.56	0.104	0.232	1.146
Support my child to participate in PA without financial expenditure	1.10	0.633	0.749	1.609	1.08	0.824	0.557	2.085
I accompany my offspring to the sports spaces on the weekend	1.00	0.909	0.673	1.423	0.68	0.324	0.309	1.475
Provide financial support for PA during working days	1.05	0.817	0.712	1.538	3.36	* 0.002	1.584	7.082
Encourage the child to be physically active during weekends	1.09	0.713	0.696	1.697	1.22	0.647	0.515	2.906
Encourage the child to be physically active during working days	1.08	0.754	0.679	1.707	1.02	0.960	0.442	2.363

PA: Physical Activity; * *p* < 0.05.

## Data Availability

Data are available by writing to fernando.rodrguez@pucv.cl in accordance with data protection law in Chile.

## Supplementary Materials

The following supporting information can be downloaded at: https://www.mdpi.com/article/10.3390/bs16091476/s1, S1: Sensitivity Analysis of the Logistic Regression Models.
