# Supplementary material for: Direct and Indirect Parental Support for Meeting Physical Activity Recommendations Among Chilean Children and Adolescents
Source: Behav Sci (Basel). 2026 Aug 25;16(9):1476. doi: 10.3390/bs16091476 (PMC13603975; doi:10.3390/bs16091476)
Supplement: Supplementary file 1 [file behavsci-16-01476-s001.zip › behavsci-4394395-supplementary.pdf]

Supplementary Material

Sensitivity Analysis of the Logistic Regression Models

Objective. To evaluate whether the maternal and paternal subsamples provided sufficient statistical sensitivity to detect associations between parental support variables and meeting the MVPA recommendation.

Methods

Rather than performing an observed post hoc power analysis, a sensitivity analysis was conducted for the binary logistic regression models. The analysis assumed a two-sided alpha level of 0.05 and 80% statistical power. The effective sample size, observed prevalence of meeting the MVPA recommendation, and empirical distribution of each parental support variable were considered. Each parental support item was analysed in an independent logistic regression model stratified by parent sex.

Sample characteristics

Subsample	Effective N	Participants meeting MVPA
Mothers	212–214	65–66
Fathers	107–108	33–34

Events per variable (EPV)

Because each parental support item was entered into a separate logistic regression model, the events-per-variable ratio remained adequate (approximately 65–66 for mothers and 33–34 for fathers). Therefore, model instability due to overfitting was unlikely. The main limitation relates to the reduced ability of the paternal subsample to detect weak associations.

Minimum detectable odds ratios (80% power)

Support variable	Mothers	Fathers
See child practising PA	1.40	1.77
Provide transport	1.37	1.58
Do PA with child	1.47	2.06
Weekend transport	1.41	1.76
Weekend PA together	1.59	2.11
Weekday PA together	1.74	2.53
Encouragement	1.36	1.54
Importance of PA	1.35	1.57
Benefits of PA	1.37	1.56
Weekend financial support	1.40	1.69
Support without money	1.52	1.84
Accompany to sport spaces	1.42	1.73
Weekday financial support	1.42	1.67
Weekend encouragement	1.57	2.03
Weekday encouragement	1.56	2.00

Interpretation

The maternal subsample was sufficiently sensitive to detect odds ratios of approximately 1.35–1.74, indicating adequate power for moderate associations. The paternal subsample was sensitive primarily to moderate-to-large associations (approximately OR 1.54–2.53). Accordingly, non-significant paternal findings should not be interpreted as definitive evidence of no association because small effects may not have been detectable.

Suggested text for the manuscript

A sensitivity analysis indicated that the maternal subsample was able to detect odds ratios ranging from approximately 1.35 to 1.74, whereas the paternal subsample could detect odds ratios ranging from approximately 1.54 to 2.53. Therefore, the paternal analyses were adequately powered to detect moderate-to-large associations but may have lacked sufficient sensitivity to identify weak associations.

Note: These estimates are based on the uploaded database and the regression strategy described in the manuscript. They should be verified against the final analytical dataset before publication.
